# Knee-Spine Syndrome: Management Dilemma When Knee Osteoarthritis Coexists With Spine Degeneration

**DOI:** 10.7759/cureus.24939

**Published:** 2022-05-12

**Authors:** Gaurav Govil, Lavindra Tomar, Pawan Dhawan

**Affiliations:** 1 Department of Orthopaedics, Max Super Speciality Hospital, Patparganj, Delhi, IND

**Keywords:** knee-spine syndrome, decision-making, total knee arthroplasty, degenerative spinal disease, total knee replacement, knee osteoarthritis

## Abstract

The elderly present with progressive degenerative osteoarthritis of the knee and lumbar spine degeneration (LSD). The coexistent conditions when presented concurrently become challenging for the clinicians and surgeons, as well as determining the predominant source of the underlying pain generation factor. The concurrent presentation of a significant knee and low back pain poses challenges in decision-making for treatment with management being normally individualized.

The review narrates the different concepts used for the assessment of knee-spine syndrome. The prime factor for the pain needs to be ascertained by evaluating the deformity of the lumbar spine and the knees to address the causative factor appropriately. A thorough history, detailed examination, and supplemental diagnostic testing will differentiate the clinical entities and guide the treatment. However, a misdiagnosis may need a secondary site surgery and further treatment to alleviate the pain. Clinicians have been challenged while differentiating between the knee and spine pathology to target prime pain generator factors for adequate pain relief, improved functional outcomes, and substantial patient satisfaction. We present our strategy for the management of knee-spine syndrome. The protocols utilized to manage the clinical scenario have been reviewed and discussed. Clinical pearls to identify and treat the symptomatic concurrent knee-spine degeneration are presented.

There is still a lack of consensus on the concurrent knee-spine degenerative pathology and its management strategy. The dilemma persists, and a case-based approach needs to be adopted by surgeons.

## Introduction and background

In the elderly, the most common musculoskeletal affection has been osteoarthritis of the joints. Arthritis may affect the knee, hip, and spine, resulting in disability, impairment, and loss of functional capacity [1]. The degeneration affects multiple joints, and the pain generation pathology contributes to an adverse quality of life. Management may include conservative and surgical procedures to alleviate pain and improve quality of life.

Any relationship changes due to any pathological or degenerative changes at the knee, hip, or spine may produce compensatory changes at other sites with the progression of deformities to maintain a balance in posture [2,3]. The relationship between the knee, hip, and spine would remain well maintained when one stands with a well-balanced posture on two feet [2,3]. The energy utilized during ambulation will be minimized when an upright posture has been maintained with well-aligned weight-bearing segments [4]. The energy spent in ambulation increases with malalignment [3,4]. The degeneration progresses to knee osteoarthritis (KOA) and lumbar spine degeneration (LSD). Knee-spine malalignment can be managed with either an arthroplasty or spinal procedures. Failures to recognize a concurrent pathology will compromise the results with an erroneous treatment [4].

Symptomatic concurrent KOA and LSD present significant challenges in management. The surgeon must contemplate whether the degeneration happens first in the spine or knee. An analogy to whether an egg came first or a chicken may not be over-justified. We lack any clear guidelines, and various management strategies have been suggested with no set pattern to follow. An area of evolving research makes it exciting and intriguing.

We reviewed the literature containing the terms “KOA,” “LSD,” and “knee-spine syndrome” from search engines PubMed and Google Scholar for articles published up to December 2021. The objective was to identify studies detailing the evaluation, assessment, and decision-making for the management of concurrent knee and spine degeneration. All studies identified by these searches were then reviewed. We excluded articles that focused on post-arthroplasty or post-spinal surgery effect on the concurrent degeneration of the spine or knee. Studies in languages other than English and those with animal or pediatric subjects were also excluded. We present a narrative of the available evidence and suggested strategies to treat the concurrent KOA and LSD pathology for its management. The review highlights the existing dilemma for clinicians’ decision-making.

## Review

An evolving scenario of degeneration

The changing dynamics and evolving scenario for KOA and LSD affection in elderly and young adults warrant a close observation of progressive and developing degenerative changes in both the knee and spine concurrently for an effective management strategy. Reduced mobility due to degenerative KOA can damage the spine [3]. Furthermore, any delay in knee arthroplasty surgery may cause irreparable LSD disease.

Knee Degeneration

The management of KOA has been studied widely, and guidelines for orthopedic surgeons have been defined based on symptomatology and radiographic correlation [5-9]. Kellgren and Lawrence have classified KOA, and it has been widely quoted for radiological assessment [10]. The study has identified the Kellgren-Lawrence (K-L) classification as a valid classification system with high inter- and intra-observer reliability and diagnostic accuracy [11]. However, they suggested using the Altman criteria to comprehensively assess the diseased state of the knee joint for diagnosing KOA [11,12].

In the evolving scenario, the rise of KOA in younger people has been surprising as the degeneration was considered to be associated with the wear and tear of the weight-bearing joints primarily due to aging [13]. Early onset of degenerative joint disease means that factors such as an unhealthy lifestyle, sporting injury, obesity, lack of exposure to the sun, and substance abuse are at play [13,14]. We are falling short on almost every front in ensuring bone health, leading to a sharp rise in KOA, even in young, active, and athletic people [14]. Degenerated knee burdens the spine in the young age group, leading to an increased propensity for LSD. Elderly KOA patients are more into pain management, unlike the young working lot, who are focused on complete and quick recovery to avoid low productivity at work.

The management of KOA in young adults responds typically to conservative measures, including exercise, weight reduction, braces, anti-inflammatory medications, intra-articular steroids, or visco-supplements [14,15]. Surgical management with an osteotomy, arthroscopy, or arthroplasty, either uni-compartmental or total knee replacement, may be reserved for poor responders depending upon the degeneration pathology and patient expectations [14,15].

The scenario in the developing world with an urban-rural divide presents more challenges in management. KOA presents with delayed or advanced stages of degeneration due to a lack of awareness about the treatment options for knee arthritis. Advanced arthritis may have a poor functional outcome [15]. The lack of societal support for the elderly or young with KOA and LSD has a psychosocial impact, limiting their social interaction capabilities [13]. The elderly develop an increased dependency and suffer from poor quality of life. Conservative management may often be counterproductive, leading to a significant burden on leading an active lifestyle. However, with a properly timed surgery, older adults could expect to reverse their inactivity, improve their quality of life, and become more active to fulfill their activities of daily living independently.

Clinical decision-making for KOA management has been complex, with radiographic parameters and functional limitations being prime criteria for replacement considerations [16]. Pain, age, obesity, and poor response to conservative management may be the other guiding factors for arthroplasty considerations [13,16].

**Figure 1:**
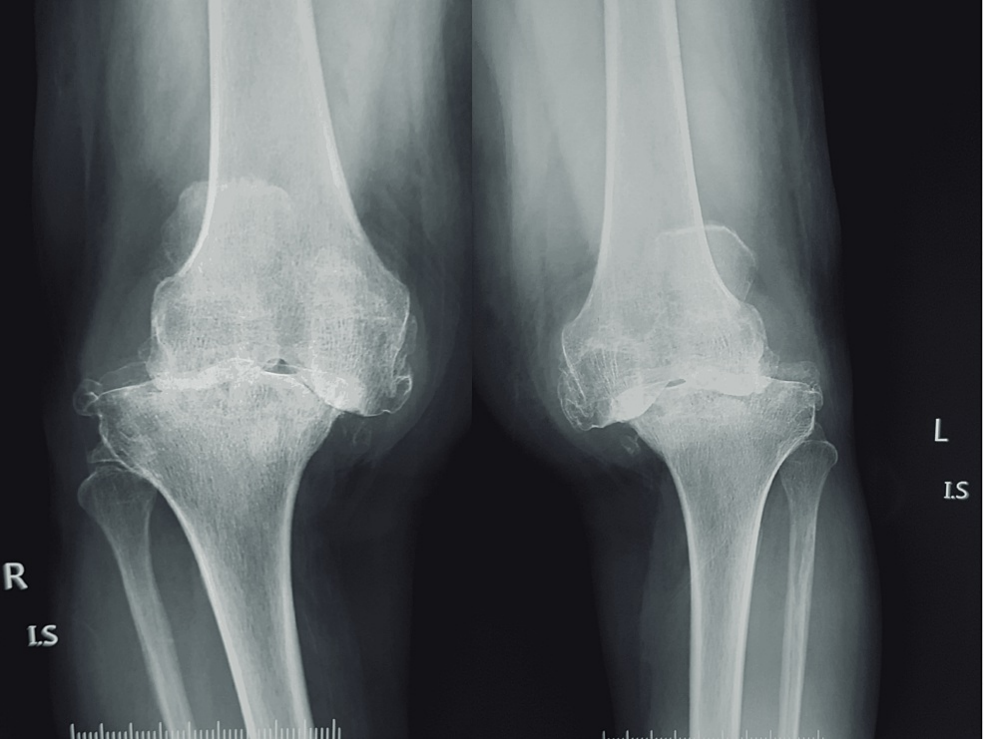
Anteroposterior radiograph of the bilateral knee joint in standing posture with grade IV osteoarthritis with large osteophytes, obliteration of joint space, subluxation of joint, and varus deformity of knees.

**Figure 2:**
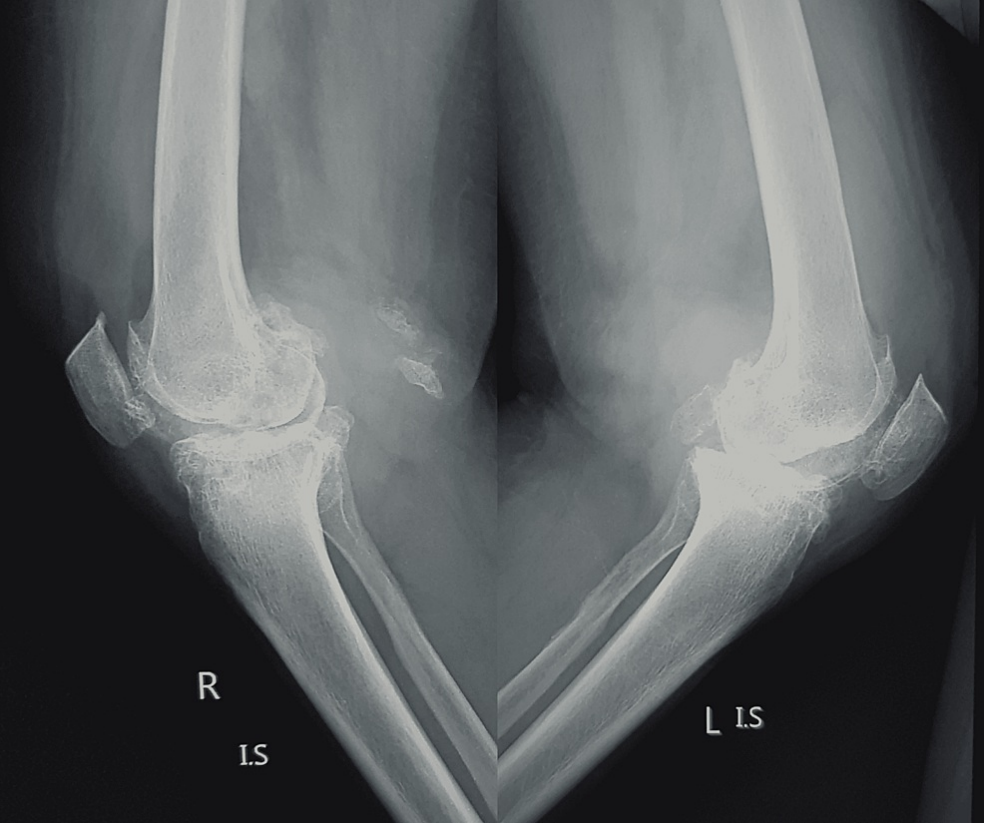
Lateral view radiograph of the bilateral knee joint with severe osteoarthritis.

A case study representing the clinical scenario for predominant KOA with coexistent LSD has been detailed. A 60-year-old female of short stature presented to the outpatient clinic with a long-standing history of pain in both knees and lower back. Initially, the onset of symptoms was mild to moderate intensity of pain in both knees. The bilateral knee pain has persisted and aggravated to severe intensity for almost two years. She now has additional complaints of severe pain in the lower back and pain in both knees. The back pain was insidious with radiation to both lower limbs and associated with heaviness and numbness in both lower limbs. The worsening pain intensity has made her confined to bed with poor ambulatory status. The conservative measures adopted for the initial management in consultation with osteopaths, naturopaths, and chiropractors for her knee pain included oral pain killers, physiotherapy, and lifestyle modifications. The clinical examination revealed severe varus deformed bilateral knees with a mild degree of bilateral knee flexion deformity. The bilateral knees have a painful arc of motion from 10 degrees to 80 degrees. The active straight leg test was positive bilaterally. The lower limbs had preserved motor function. However, paresthesia presented in both lower limbs. She was mildly hypertensive and diabetic, with a BMI of 30, with no other known significant personal history. The plain radiograph for both knees revealed K-L grade IV changes (Figures 1, 2).

**Figure 3:**
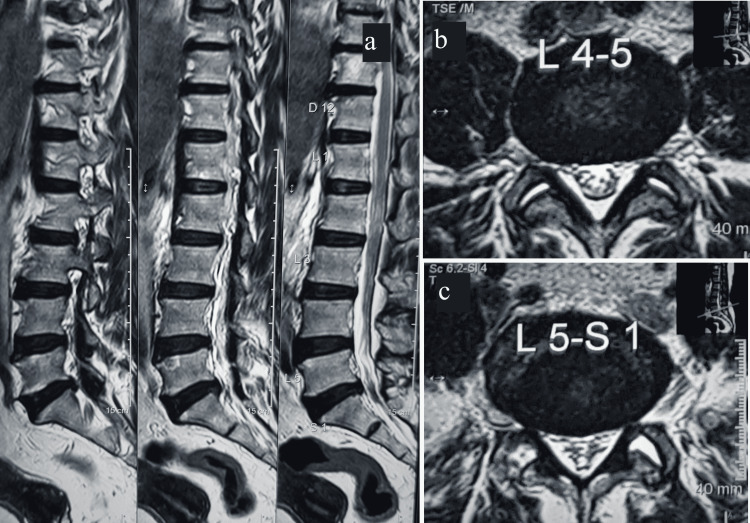
Magnetic resonance imaging of the lumbar spine showing discoid degenerative changes in sagittal section (a), nerve root compression at L4-L5 level (b), and nerve root compression at L5-S1 disc level (c).

The magnetic resonance imaging of the lumbar spine revealed spondylosis, discoid degenerative changes with lumbar osteophytes, facetopathy, variable disc desiccation, and nerve root encroachment by L5/S1 disc causing compression (Figure 3).

The nerve conduction velocity test suggested S1 radiculopathy bilaterally. There was a dilemma in the management strategy on whether to plan for knee arthroplasty first in the presence of the significant knee deformity with persisting radiculopathy or plan spine decompression first for a high VAS score and progressive restriction of activities of daily living. The resultant functional knee outcome gets unpredictable. The predominance of painful bilateral knees with surgically correctable varus knee deformity in the absence of any motor deficit of the lower limb without neurogenic claudication guided our decision for bilateral knee arthroplasty first.

Spine Degeneration

Degeneration of the intervertebral disc with resulting back pain in the elderly and young is multifactorial. The result leads to chronic instability and functional disability [17]. Progression of degeneration from asymptomatic to symptomatic affection occurs in 10% of males under 50 years and up to 50% affection for above 70 years. Progressive LSD leads to secondary spinal stenosis, spondylolisthesis, and compression neuropathies [17]. A meta-analysis performed for the prevalence of LSD disease evident on magnetic resonance imaging concluded that individuals with back pain have a higher prevalence of degeneration than asymptomatic ones in adults 50 years or younger [18].

In the evolving scenario, the rise of LSD in younger people has been surprising. The rising incidence in young adults will eventually manifest LSD disease in the elderly [18]. Early onset of spinal disorder includes disc desiccation, disc prolapse, disc ruptures, and spinal muscular imbalance that contributes to progressive degeneration. The risk factors commonly associated include a sedentary lifestyle, poor exercise scheduling, and imbalance of the spinal musculature.

The management for LSD presenting with low back pain and radiculopathy initially focuses on nonsurgical, conservative measures. Pain medications, physical therapy, and modulation of lifestyle changes are recommended. Persisting or poor responders may require interventional modalities inclusive of epidural steroid injections, transforaminal injections, and nerve root blocks. However, managing symptomatic unresolved radicular pain, neurological compromise, and claudication has been complex and has become the guiding factor for spine surgery considerations [19-22].

**Figure 4:**
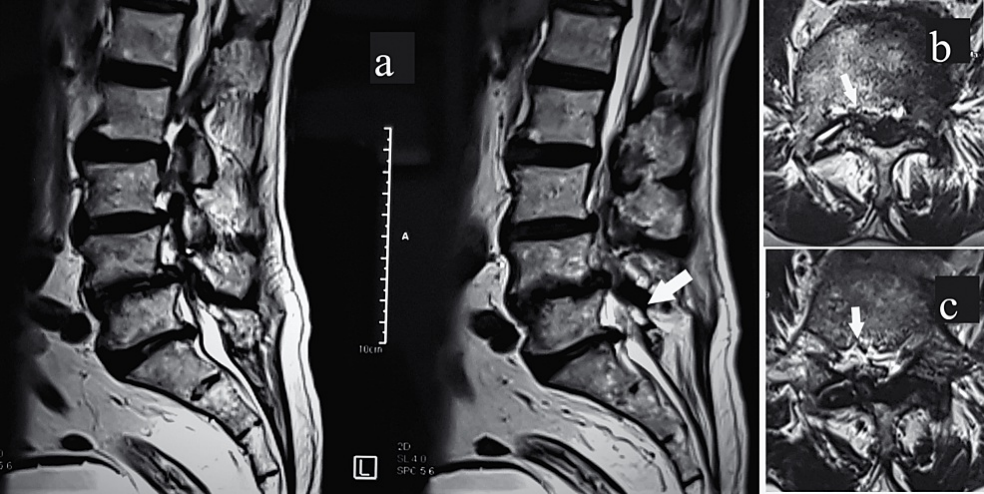
Magnetic resonance imaging of the lumbar spine showing marked spondylo-discoid degenerative changes in sagittal section (a) and canal stenosis at L3-L4 level (b) and L4-L5 disc level (c).

A case study that represents the clinical scenario for predominant LSD with coexistent KOA has been detailed. A 65-year-old male presented to the outpatient clinic with multiple symptomatic episodes of pain in the lower back of a long-standing duration of about 20 years. They have been managed with conservative measures, including restricted activities, anti-inflammatory medications, physiotherapy, and spine strengthening exercises. There has been persisting lower back pain for almost three years with increasing difficulty in doing daily routine activities. The knee pain has been gradual in onset, progressing to disabling pain for almost two years. The heaviness in both lower limbs aggravates when standing for prolonged periods. Lying supine gives comfort; however, ambulation increases pain and requires support. Pain dissuades him from attempting to walk for his normal routine activities. Clinical examination revealed loss of lordosis with pelvic tilting. The motor evaluation was standard. Deep tendon reflexes were diminished, and marked paresthesia was presented in both lower limbs. Bilateral knees were tender on palpation, and crepitus was present with a painful arc of motion. The ambulation status with support mobilization was painful. The magnetic resonance imaging of the lumbar spine revealed secondary canal stenosis for the lumbar segment from L3 to S1 with spondylo-discal degenerative changes, facetopathy, and nerve root compression (Figure 4).

**Figure 5:**
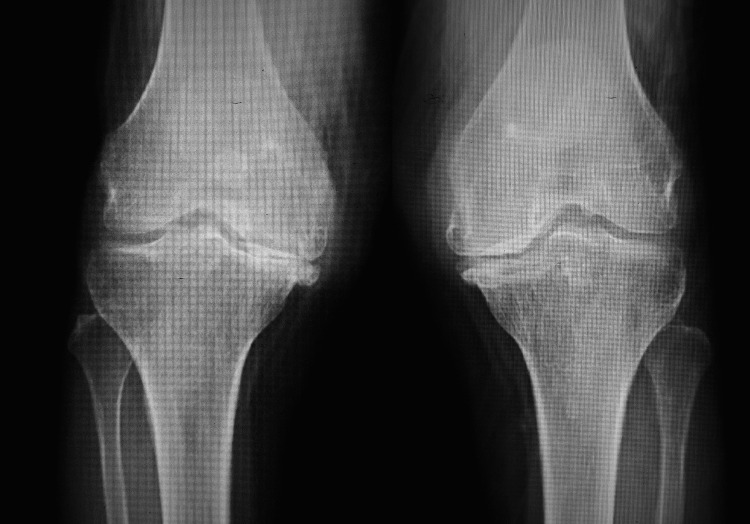
Anteroposterior radiograph of the bilateral knee in standing posture with grade IV osteoarthritis with large osteophytes, narrowing of joint spaces, marked sclerosis of the medial joint, and varus deformity of both knees.

**Figure 6:**
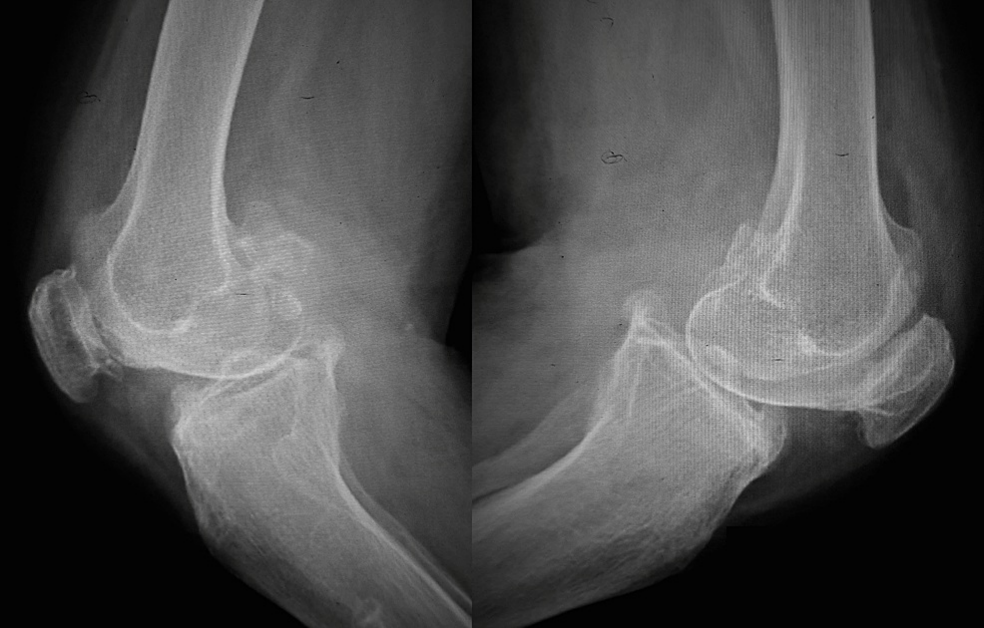
Lateral view radiograph of the bilateral knee joint with severe osteoarthritis.

The nerve conduction velocity test suggested L4 and L5 radiculopathy bilaterally. The plain radiograph for both knees revealed K-L grade IV changes (Figures 5, 6).

There was a dilemma in the management strategy on whether to plan for spine decompression surgery first in the presence of the aggravating knee pain or plan knee arthroplasty first. The intra-articular installation gave partial relief from knee pain for the initial two weeks; however, neurogenic pain persisted with marked difficulty in doing his daily routine activities. The predominance of chronic lower back pain with resultant neurogenic claudication even in the presence of bilateral KOA of severe grade guided our decision in favor of spinal decompression surgery first.

Discussion

The literature review for KOA and LSD from electronic databases with evidence and suggested strategies to treat concurrent KOA and LSD pathology for its management have been presented.

Offierski et al. (1983) [23] studied and presented their observation of 35 elderly for concurrent hip and spine pathology to define “hip spine syndrome.” To differentiate the origin of the pain in complex hip spine syndrome, they advocated lignocaine infiltration into the hip joint, and assessment of pain relief was considered an indication of hip pathology as the origin of the pain. Similarly, nerve root infiltration along the lumbar spine at the L4 level allowed localization of the source of pain, and if significant improvement is present, there will be a requirement for spine pathology management.

Hip flexion deformity leads to forward pelvic tilting with compensatory exaggerated lumbar lordosis and progression to spine degeneration. A fixed adduction deformity of the hip leads to pelvic obliquity and progression to lumbar scoliosis with nerve root compression symptomatology. The correction of deformity of the hip changes biomechanics to reduce and correct the secondary spinal deformity. The authors concluded that precise assessment to recognize the source of the pain would necessitate an appropriate treatment plan with a low probability of misdiagnosing and mismanaging concurrent pathology.

Itoi (1991) [2] was the first to report an interrelationship between thoracic kyphosis spinal malalignment and flexion of the knee in an osteoporotic spine by analyzing the lateral radiographic view of the spine. The study on spinal osteoporotic patients compensated an increase in thoracic kyphosis with an increased lumbar lordosis. When the lumbar spine could not compensate due to a lower spine interface, posterior tilting of the sacrum until it is absorbed in the sacroiliac joint, followed by further posterior pelvic tilt, this compensation, once it reaches a maximum limit, causes inclination of the hip and femur, followed by the development of knee flexion deformity. Clinically, lower thoracic kyphosis was unfavorable and observed to aggravate low back pain.

Tsuji et al. (2002) [24] classified concurrent hip and spine pathologies into certain degrees of involvement into a simple, secondary, and complex syndrome. They discussed “hip-spine syndrome” first described by Offierski and MacNab. Their study further examined the effect of changes in lumbar lordosis and sacral inclination on the knee. The measures examined were patellofemoral pain. They correlated that decreased lumbar lordosis and sacral inclination lead to increased knee flexion on standing and increased thigh muscle tension. The study was based on the Japanese population. Degenerative spine changes and patellofemoral pain coexisted, which is called “knee-spine syndrome.”

Interestingly, as a contradiction, Murata et al. (2003) [25] detailed the “hip-spine syndrome” of Jackson and McManus who based their observations on the measurements for lumbar lordosis and sacral inclination concerning the degree of the extension of the hips. They further studied the knee extension measurement effect on lumbar lordosis and sacral inclination based on a spinal radiographic evaluation without radiographic evaluation of KOA changes. The correlation between knee extension and lumbar lordosis and the causation of degenerative changes to the spine was called “knee-spine syndrome.” The study was again based on the Japanese population.

In their review article, Devin et al. (2012) [26] identified clinical history, detailed physical examination, and diagnostic techniques to ascertain the primary pain generator pathology in hip-spine syndrome. They concluded that an inappropriate methodology might lead to misdiagnosis with inadequate pain relief and poor patient satisfaction.

Harato et al. (2008) [27] hypothesized that knee flexion contracture would lead to spinal imbalance. They postulated that imbalance might contribute significantly to the manifestation of knee spine syndrome. They identified static and dynamic changes in trunk equilibrium in 10 healthy patients who had no knee or spine pathology evidence. Any pathological spine or knee degeneration may present a varying degree of symptomatology in clinical practice. They postulated that the implications of spinal deformity in the causation of knee degeneration or vice versa might need thorough clinical evaluation through a detailed history, comprehensive examination, and radiographic imaging of the affected joints to formulate an effective management strategy.

Tauchi et al. (2015) [28] were the first to study the role of spinal factors inclusive of spinal alignment and spinal range of motion as a risk factor for the causation of KOA in a Japanese population group of 170 elderly. The study identified various other known risk factors for KOA. “Elderly,” “obese” “females” with “lower bone mineral density” are prone to KOA. Those with a history of smoking, genetic predisposition, and previous knee injury were additional independent risk factors for KOA progression. They evaluated KOA radiographically utilizing the K-L classification for KOA and used a spine mouse, a computer-aided, noninvasive device for measuring spinal alignment and movements. They concluded that an increased sacral inclination angle was an independent risk factor for the causation of KOA. They disregarded the finding of the previous study for thoracic kyphosis angle association in the causation of KOA and emphasized lumbar lordosis and spinal movements as more significant factors for the causation of changes in sacral inclination. However, the solid positive speculation between an abnormal spinal posture and overloading of the knee in flexion remained unclear regarding which factor between the spine and the knee occurred first.

In their prospective study, Modi et al. (2019) [29] evaluated 10 operated patients with knee spine syndrome who had undergone either a total knee replacement or a spinal fixation for management. They formulated diagnostic and management algorithms for knee spine syndrome patients. The grading of KOA was done using the K-L classification. Their assessment of the severity of claudication and knee pain symptomatology ascertained their line of management whether to operate on the spine or knee first. They operated on the spine in six and the knee first in four patients, with secondary procedures done in six patients. They emphasized that spinal decompression is necessary for the presence of radiculopathy and claudication for pain relief. They also suggested spinal biomechanics, spine de-loading, and improved pain in the back after correcting knee deformity and alignment by arthroplasty in cases presenting predominantly with back pain. They identified stress fracture with the inability to bear weight as a red flag sign for advocating knee replacement first in such patients. They also advocated neurological compression with bladder bowel involvement as a red flag sign for operating the spine first for management. They concluded that the surgeon’s clinical judgment might be the primary guiding factor for decision-making to avoid staged procedures.

In their study, Oshima et al. (2019) [3] focused on the knee-hip-spine syndrome, emphasizing posture correction. They detailed the compensatory detrimental effects of postural changes arising from the degeneration of either the knee, hip, or spine with the advancement of the degeneration in the nonaffected or uninvolved part. The postural malalignment due to degeneration increases the tendency for falls in the elderly, especially osteoporotic, leading to fractures with increased mortality and morbidity. A sagittal vertical axis is an essential index of stable standing posture with any alteration affecting quality of life indices. They emphasized that the sagittal vertical axis increases with age, leading to reduced back muscle strength and limited spine motion.

Kim et al. (2020) [4] studied and evaluated radiological and clinical changes in an operated patient with LSD with subsequent development of an advanced KOA. They conducted a retrospective comparative clinical study using time-dependent outcome analysis for sagittal spinal alignment following posterior spinal instrumentation and fusion. They concluded that clinical outcomes following a spinal fixation with lumbar fusion deteriorated with the onset of KOA degeneration. Adult spinal deformity that presents with low back pain and variations in spinal alignment leads to compensatory mechanisms affecting pelvic inclination. An increased negative influence on clinical outcomes with an increased grading of degenerative KOA was noted.

Goodman et al. (2020) [30] conducted a survey based on clinical scenarios of concurrent and advanced KOA and LSD disorders. They concluded that the severity and type of deformity might influence the preference of treatment order. The survey was conducted among knee arthroplasty and spine surgeons with more than 25 years of experience. They sought their preferred treatment order and comments. Although limited by around 50% responses, lacking the objective assessment for knee deformity, and liable to readers’ extrapolation of problems presented, the study emphasized individualized and shared decision-making as the mainstay. “Spine surgery first” in the absence of deformity of the knee, while “knee surgery first” in the presence of severe knee deformity was advocated.

Author strategy in the management of KOA and LSD

**Figure 7:**
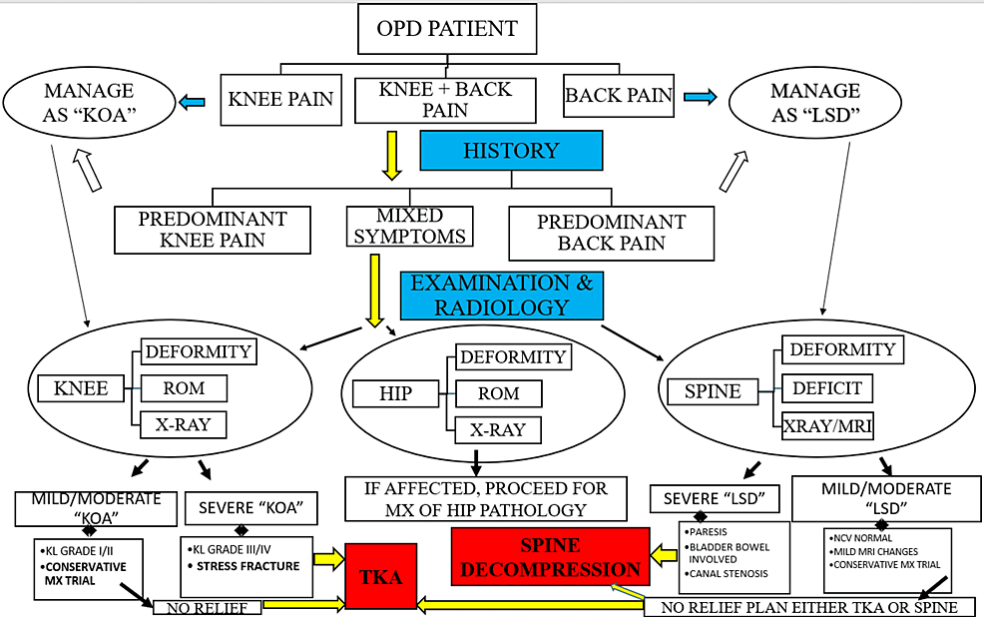
Flowchart for knee spine syndrome management strategy.

A thorough history-taking with meticulous examination aided by radiological evaluation will initially guide the appropriate management strategy. Imaging modalities, nerve conduction studies, and local injection installation techniques differentiate the primary pain pathology between the knee, hip, and spine. The strategy adopted for the management of coexistent knee and spine degeneration is presented in a flowchart in Figure 7.

The clinician needs to identify any red flag signs for the knee and spine [29]. A thorough assessment of knee and spine deformities further guides the preference of management. The vicious cycle ensues with a progressive knee flexion deformity, which alters the spinal alignment [27]. The disbalance leads to the progression of LSD. Lower back pain symptoms with radicular pain may aggravate and worsen the ambulatory status. Correction of the knee alignment during arthroplasty may avoid the worsening or progression of spinal degeneration. Similarly, the correction of spinal alignment during spinal surgeries may lessen knee loading and prevent the progression of degeneration.

A preoperative radiological evaluation for three sites should be done for an elderly planned for knee arthroplasty or spine surgery. The assessment may guide in identifying the concurrent degeneration. Review studies have focused on preoperative evaluation by knee radiographs before knee arthroplasty. Radiological techniques evaluate axial, sagittal, and coronal planes for angle, axis, and index measurement [31]. The three sites include the pelvis with both hips in anteroposterior view, lumbosacral spine in lateral view, and bilateral knee in anteroposterior view in standing posture or a hip-knee-ankle long film of the lower extremity, to identify any significant contributing degeneration of any other site [32]. The outcome can be prognosticated before arthroplasty, and the likely need for a secondary procedure can be discussed [33,34].

Limitations

This narrative review aimed to highlight the evidence in the literature for researchers to improve their KOA and LSD management strategies. The suggested strategy has limitations as it presently lacks the support of statistical evidence. The review does not detail postoperative evaluation after a knee arthroplasty or spine surgery. The evaluation of clinical and functional outcomes may further guide researchers in managing concurrent knee-spine degeneration. They can subsequently base their future studies on assessing knee-spine imbalance.

## Conclusions

Whether to operate on the knee or spine first, the preference of surgery needs clinical correlation, and it continues to puzzle even seasoned clinicians and surgeons. No sufficient evidence is available to guide the choice of treatment order for the symptomatic patient group. An astute clinical judgment following a thorough physical examination and radiological assessment remains the guiding factor for managing concurrent knee-spine degeneration.

The dilemma of decision-making remains unanswered mainly, and challenges persist. Further studies are required to understand the management of concurrent knee and degenerative spine disorders in the elderly.
